# Detection and Differentiation of Threonine- and Tyrosine-Monophosphorylated Forms of ERK1/2 by Capillary Isoelectric Focusing-Immunoassay

**DOI:** 10.1038/srep12767

**Published:** 2015-08-03

**Authors:** Inga Kraus, Daniela Besong Agbo, Markus Otto, Jens Wiltfang, Hans Klafki

**Affiliations:** 1 LVR-Hospital Essen, Department of Psychiatry and Psychotherapy, Faculty of Medicine, University of Duisburg-Essen, Essen, Germany; 2 German Center for Neurodegenerative Diseases (DZNE), Research Site Goettingen, Germany; 3 Department of Neurology, University of Ulm, Germany; 4 Dept. of Psychiatry and Psychotherapy, University Medical Center Goettingen (UMG), Georg-August-University Goettingen, Germany

## Abstract

The extracellular signal regulated kinases ERK1/2 play important roles in the regulation of diverse cellular functions and have been implicated in several human diseases. In addition to the fully activated, diphosphorylated ERK1/2 protein, monophosphorylated forms of ERK1/2 have been observed, which may have distinct biological functions. We report here on the highly sensitive detection and differentiation of unphosphorylated, threonine-phosphorylated (pT), tyrosine-phosphorylated (pY) and diphosphorylated ERK1 and ERK2 by capillary isoelectric focusing followed by immunological detection (CIEF-immunoassay). Eight different phosphorylated and unphosphorylated forms of ERK1/2 were resolved according to charge. The unequivocal identification and differentiation of ERK1 and ERK2 forms monophosphorylated at either threonine or tyrosine was achieved by competitive blocking with specific phospho-peptides and different phosphorylation-sensitive antibodies. The suitability of the additional pT-ERK1/2 and pY-ERK1/2 differentiation for the time-resolved in-depth study of phospho-form distribution in response to specific stimuli is demonstrated in human neuroblastoma SH-SY5Y and monocytic THP-1 cell lines, and in human peripheral blood mononuclear cells.

The extracellular regulated protein kinases ERK1 and ERK2 are closely related members of the mitogen activated protein kinase (MAPK) family and are involved in the regulation of diverse cellular functions including proliferation, differentiation, cell adhesion, cell cycle progression, survival and apoptosis (for reviews, see^1^,^2^,^3^,^4^,^5^). In the central nervous system, ERK1/2 signalling plays important roles in synaptic plasticity, long-term potentiation, long-term depression and cell survival (for reviews, see^6^,^7^,^8^).

Dysregulation of MAPK signalling pathways has been implicated in diverse human diseases including different types of cancer, Alzheimer’s disease, Parkinson’s disease and amyotrophic lateral sclerosis (ALS) (for reviews, see^9^,^10^). ERK1 and ERK 2 are activated by dual phosphorylation by the upstream kinases MEK1/2 at a conserved threonine-glutamate-tyrosine (TEY) motif (Thr 202 and Tyr 204 in human ERK1, Thr 185 and Tyr 187 in human ERK2)^2^,^11^. While phosphorylation at both, Tyr and Thr is required for full enzyme activation, also the monophosphorylated forms of ERK2 were shown to have appreciable kinase activities *in vitro* at 1 mM ATP^12^, which is in the range of physiological intracellular ATP levels in mammalian cells^13^. Thus, monophosphorylated forms of ERK2 were proposed to possibly represent intermediate activity states which might have distinct biological roles *in vivo*^12^. Tyrosine-monophosphorylated ERK2 was reported to transiently associate with the Golgi complex in HELA cells during cell cycle G2 and M phases, suggesting a role in the regulation of Golgi structure^14^.

High resolution capillary isoelectric focusing with immunological detection and chemiluminescent readout was introduced in 2006 as a novel technology allowing for the separation and highly sensitive immunological detection of unphosphorylated, monophosphorylated and doubly phosphorylated ERK1/2 isoforms^15^.

We report here that additionally, Tyr- and Thr-monophosphorylated pY-ERK1/2 and pT-ERK1/2 can be resolved and differentiated by this technology. For an unequivocal assignment of the respective peaks we used different antibodies against phosphorylated ERK1/2 and performed competitive blocking experiments. For a proof of principle, the method was finally applied to the high resolution analysis of ERK1/2 phosphorylation in bradykinin-stimulated SH-SY5Y cells and N-methyl-D-aspartate (NMDA)-treated human peripheral blood mononuclear cells (PBMCs).

## Results

### Analysis of different phosphorylation forms of ERK1 and ERK2 in stimulated THP-1 and SH-SY5Y cells

**Figure 1:**
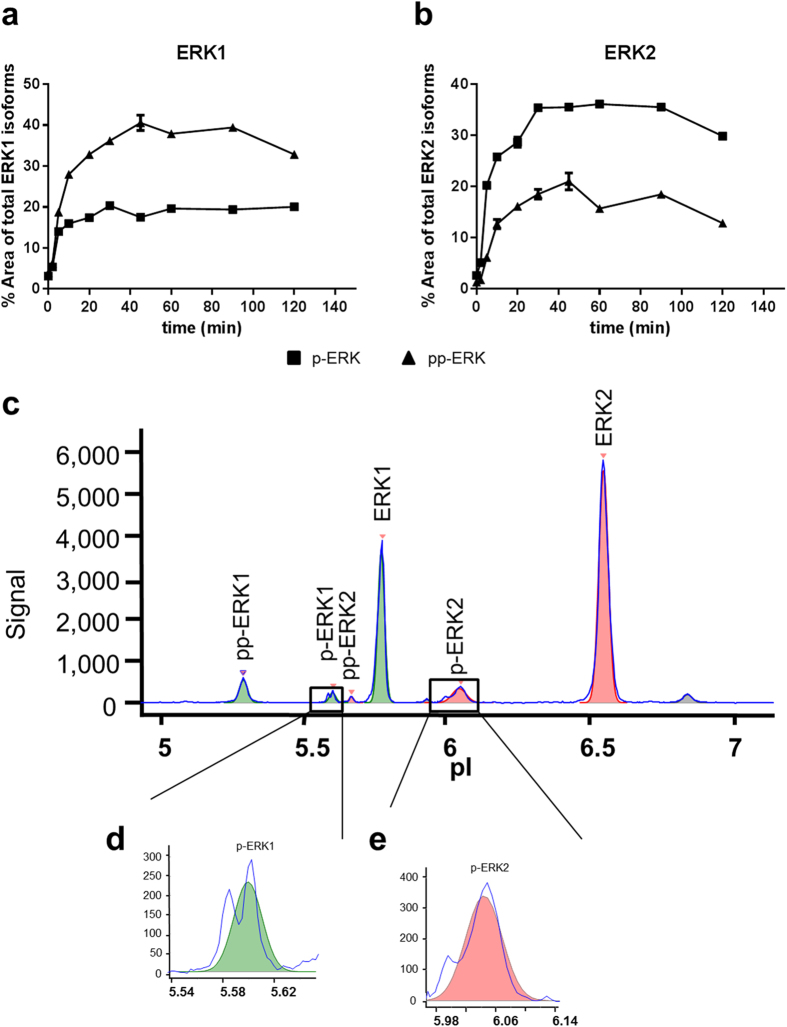
Occurrence of mono- and diphosphorylated ERK1 and ERK2 in PMA-activated THP-1 cells. THP-1 cells were treated with 200 nM PMA and lysed after different incubation times (0, 2, 4, 8, 10, 15, 30, 45, 60, 90, 120 min). Lysates were subjected to CIEF-immunoassay with a pan-ERK antibody (ProteinSimple). The relative peak areas of mono- and diphosphorylated ERK1 (**a**) and ERK2 (**b**) isoforms are shown. Squares represent the monophosphorylated and triangles the doubly phosphorylated ERK1/2 isoforms (mean ± SD from technical duplicates). A representative electropherogram after 2 min of PMA stimulation is shown in (**c**). Detail enlargements demonstrate that the signals corresponding to monophosphorylated ERK1 (p-ERK1) (**d**) and ERK2 (p-ERK2) (**e**) appeared as two distinct peaks each, which were not fully resolved.

Human monocytic (THP-1) cells were stimulated with Phorbol 12-myristate 13-acetate (PMA), lysed after different incubation times and subjected to CIEF-immunoassay with a phosphorylation-insensitive pan-ERK antibody. A rapid and persistent occurrence of di- and monophosphorylated ERK1/2 isoforms was observed (Fig. 1a,b). Maxima in phosphorylation were reached after approximately 30 to 45 min. Concerning ERK1, the diphosphorylated isoform was the predominant one, whereas monophosphorylated ERK2 was more abundant than diphosphorylated ERK2. A representative electropherogram of a cell lysate prepared after 2 min treatment with PMA is shown in Fig. 1c. The assignment of the observed peaks was done according to the original report on the technology (15, see Supplementary Information, Fig. S1). Upon closer inspection of the electropherograms, we noticed that the monophosphorylated ERK1 and ERK2 signals after relative short incubation with PMA regularly appeared as two peaks that were not fully resolved from each other (Fig. 1d,e).

**Figure 2:**
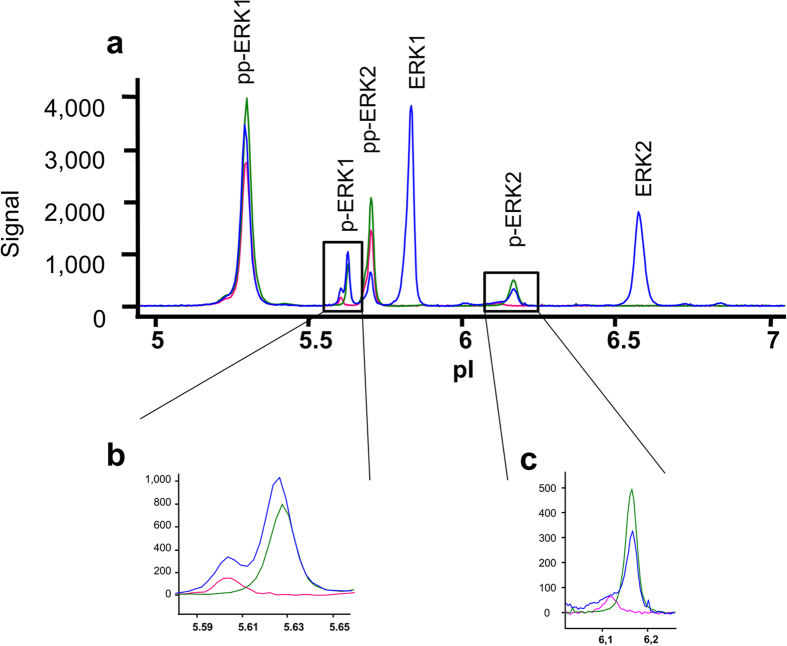
Resolution of the monophosphorylated ERK1 and ERK2 double peaks with different phospho-antibodies. SH-SY5Y cells were treated with 100 nM bradykinin for 4 min and lysates were subjected to CIEF-immunoassay (**a**). Immunodetection was performed with the phosphorylation-insensitive pan-ERK Ab PS#040-474 (blue curve), with the phospho-ERK Ab CS#4370 (green curve) and with phospho-ERK Ab CS#4377 (pink curve). The enlargements clearly reveal distinguishable double peaks for the p-ERK1 (**b**) and p-ERK2 (**c**) area and show that the two phospho-antibodies display different selectivities.

To assess whether this finding could be reproduced in a different cell line and with a more physiological ERK1/2 signalling cascade activator, we treated SH-SY5Y cells with bradykinin. Representative electropherograms after 4 min incubation time and probed with a phosphorylation-insensitive pan-ERK1/2 antibody (blue curve) and two different anti phospho-ERK1/2 antibodies (green and pink curves) are shown in Fig. 2a. In line with the observations from PMA-treated THP-1 cells (see above), detection with anti-pan-ERK produced two peaks for monophosphorylated p-ERK1 and p-ERK2, each. Importantly, the two anti-phospho-ERK1/2 antibodies tested here displayed different selectivities for the presumed monophosphorylated ERK1 and ERK2 species: The monoclonal rabbit antibody “Phospho-p44/42 MAPK (Erk1/2) (Thr202/Tyr204) (197G2) Rabbit mAb #4377 (Cell Signaling)” selectively recognized the more acidic p-ERK1 and p-ERK2 peaks, respectively. In contrast, the monoclonal rabbit antibody “Phospho-p44/42 MAPK (Erk1/2) (Thr202/Tyr204) (D13.14.4E) XP® Rabbit mAb #4370 (Cell Signaling)” showed preference for the more alkaline p-ERK1 and p-ERK2 peaks. Similar results were obtained with human PBMCs freshly prepared from whole blood and stimulated with PMA or with NMDA in combination with glycine (data not shown).

### Unequivocal identification of pT- and pY-monophosphorylated ERK1/2 isoforms by competitive blocking

**Figure 3:**
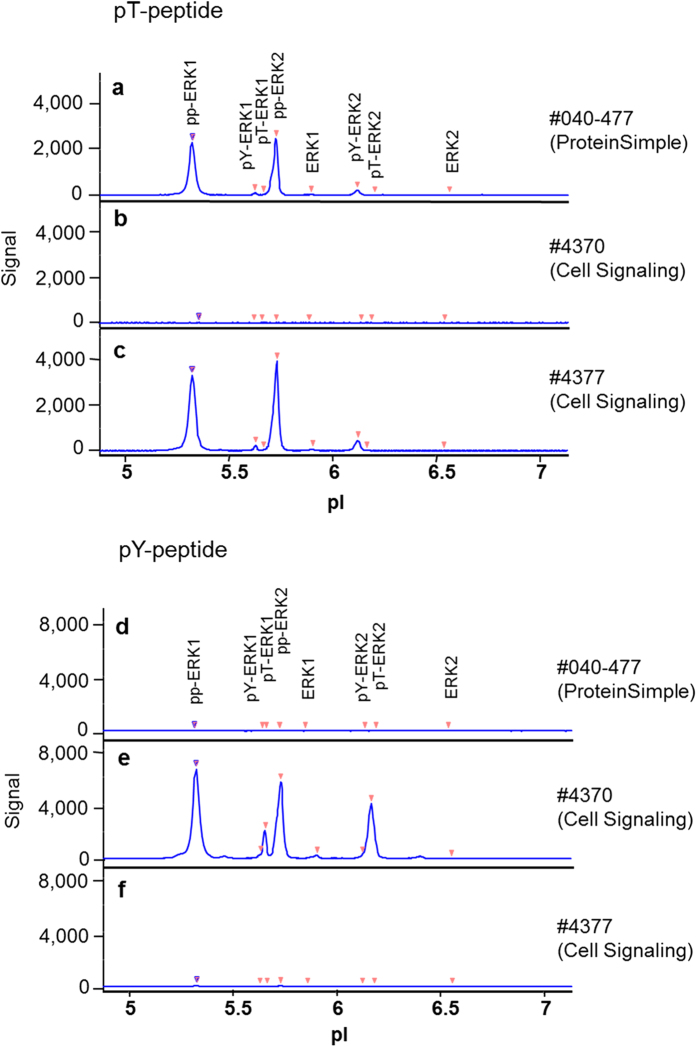
Competitive blocking of specific antibody binding by synthetic peptides. A lysate of PBMCs stimulated with 200 nM PMA for 10 minutes served as a sample. The peptides were pre-diluted, mixed with antibodies each and applied to CIEF-immunoassay as described in materials and methods. We used 10 μg/ml of the pT-peptide and 50 μg/ml of the pY-peptide to achieve an optimal blocking of the antibodies. The pT-peptide was mixed with #040-477 (**a**), #4370 (**b**) and #4377 (**c**). According to this, the pY-peptide was also mixed with #040-477 (**d**), #4370 (**e**) and #4377 (**f**).

To serve as an appropriate sample with high levels of phosphorylated forms of ERK1 and ERK2, human PBMCs derived from whole blood of a healthy volunteer were treated with 200 nM PMA for 10 min. For competitive blocking experiments, the following synthetic peptides (kindly provided by Dr. Borek Vojtesek, Moravian Biotechnology, BRNO, Czech Republic) encompassing the ERK1/2 activation motif were used: (I) unphosphorylated KTGFLTEYVATR, (II) doubly phosphorylated KTGFL(pT)E(pY)VATR (pp-peptide), (III) pT-monophosphorylated KTGFL(pT)EYVATR (pT-peptide) and (IV), pY-monophosphorylated KTGFLTE(pY)VATR (pY-peptide). Note that the amino-terminal lysine residue (K) was added for coupling purposes in a different context. Each one of the four different peptides was individually pre-mixed with three different phospho-ERK antibodies prior to the CIEF-immunoassay: #040-477 (ProteinSimple), #4370 (Cell Signaling), #4377 (Cell Signaling). Pre-incubation of any of the three tested anti-phospho-ERK1/2 antibodies with the pp-peptide completely abolished the signals in the CIEF-immunoassay (data not shown). The pT-peptide blocked immunodetection of mono and diphosphorylated ERKs by antibody #4370 but not by antibody #4377 or #040-477 (Fig. 3a–c). In contrast, the pY-peptide abolished the immunodetection of mono- and diphosphorylated ERK1 and ERK2 by antibody #4377 and #040-477 (Fig. 3d–f). These findings indicate that antibody #4370 detects doubly phosphorylated and pT-monophosphorylated ERK1 and ERK2 while antibodies #4377 and the #040-477 recognise diphosphorylated and pY-monophosphorylated ERK1/2. These antibodies thus allow for an unequivocal identification of the different monophosphorylated ERK1 and ERK2 peaks. Accordingly, the more acidic of the monophosphorylated p-ERK1 and p-ERK2 peaks can be assigned to pY-ERK1 and pY-ERK2 and those with slightly more alkaline observed pI-values to pT-ERK1 and pT-ERK2, respectively. The unphosphorylated peptide served as a control and had no effect on specific antibody binding (data not shown).

### Time dependent occurrence of pY- and pT-ERK1/2 forms in stimulated SH-SY5Y cells and PBMCs from human whole blood

**Figure 4:**
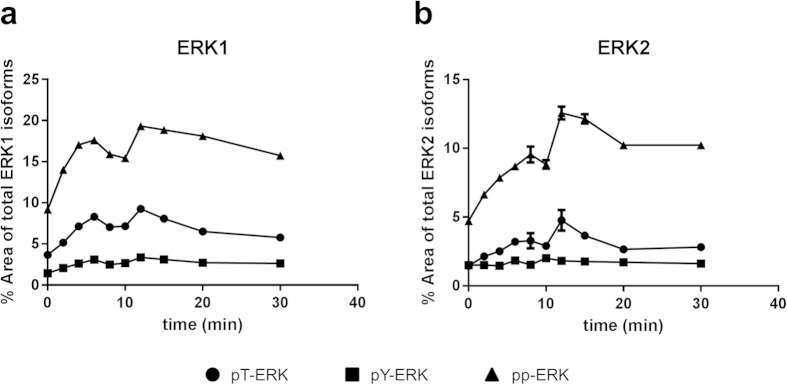
Time course of BK-stimulated SH-SY5Y cells with additional differentiation of 6 phospho-species altogether. SH-SY5Y cells were stimulated with 100 nM bradykinin for different incubation times (0, 2, 4, 6, 8, 10, 12, 15, 20, 30 min), lysed and applied to CIEF-immunoassay with the pan-ERK Ab #040-474 (ProteinSimple). The relative abundances (means ± SD of technical duplicates) of mono- and diphosporylated ERK1 (**a**) and ERK2 (**b**) are displayed in percent of total ERK1 and total ERK2 signals, respectively. Dots represent threonine-monophosphorylated ERK1/2, squares the tyrosine-monophosphorylated ERK1/2 and triangles diphosphorylated ERK1/2. One representative out of five independent experiments is shown.

As the phosphorylation-insensitive pan-ERK antibody (ProteinSimple) detects all ERK isoforms including all phosphorylated ones (Fig. 2), experiments on the kinetics of ERK1/2 phosphorylation with a specific focus on the threonine- and tyrosine-monophosphorylated forms can be performed with this single antibody in the CIEF-immunoassay. This enables a direct relative quantification of all different phosphorylated and unphosphorylated ERK1 and ERK2 signals in the same capillary. So we next applied the new possibility to differentiate between pY- and pT-monophosphorylated ERK1 and ERK2 to a more detailed analysis of time-dependent ERK1/2 phosphorylation states in SH-SY5Y cells treated with 100 nM bradykinin (BK). The incubation was stopped after different times, cells were lysed and these samples were subjected to CIEF-immunoassay. Figure 4 shows one representative out of five independent experiments. As it turned out, in bradykinin-treated SH-SY5Y cells, doubly phosphorylated pp-ERK1 and pp-ERK2 were more abundant than the different monophosphorylated forms at all tested time points. In this representative experiment, maximum relative abundancies of approximately 20% of pp-ERK1 (Fig. 4a) and 13% of pp-ERK2 (Fig. 4b) were observed after 12 min. The pT-ERK1 and pT-ERK2 forms clearly exceeded pY-ERK1 and pY-ERK2, respectively. Tyrosine-monophosphorylated ERK1/2 appeared to represent only a minor species under these experimental conditions. The occurrence of mono- and diphosphorylated ERK1 and ERK2 followed a biphasic time course: The first maximum of ERK1 activation was reached after 6 min, followed by a slight decrease until 10 min and a second maximum at 12 min which was then followed by a continuous but slow dephosphorylation. The phosphorylation kinetics for ERK2 were similar. The first maximum was reached after 8 min incubation time followed by a slight decrease and a second maximum after 12 min. As mentioned above, the experiment was repeated five times with similar outcome. In all experiments, we observed biphasic ERK1/2 activation, with maxima at the same time points and with pp-ERK1/2 always being the predominant isoform, followed by pT-ERK1/2 and pY-ERK1/2. However, the calculated percentage areas of the different phospho-ERK1/2 forms at the different time points varied between the individual experiments to some extent.

**Figure 5:**
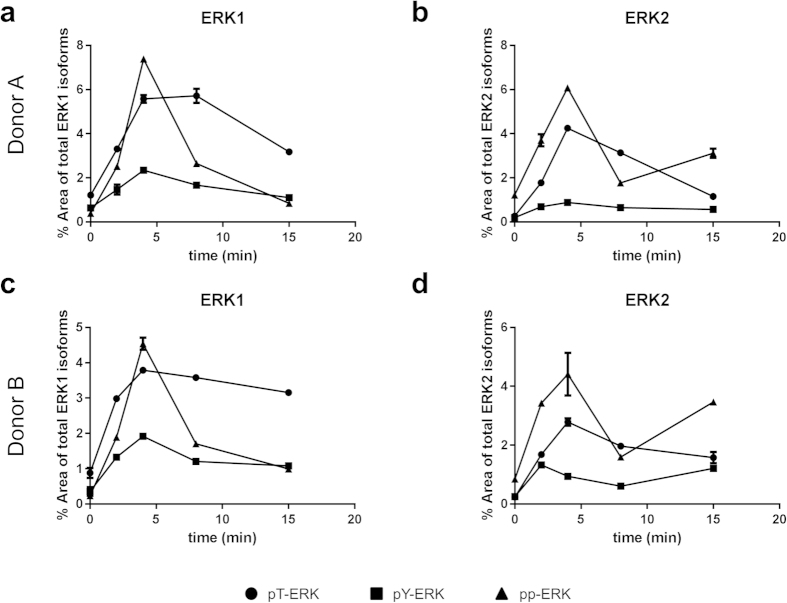
PBMCs derived from human whole blood were treated with 50 μM NMDA in combination with 10 μM glycine for different incubation times (0, 2, 4, 8, 15 min), lysed and subjected to CIEF-immunoassay with the pan-ERK antibody #040-474 (ProteinSimple). The relative abundances (means ± SD of technical duplicates) of mono- and diphosporylated ERK1 (a + c) and ERK2 (b + d) are displayed in percent of total ERK1 and total ERK2 signals, respectively. (a + b) belong to donor A and (c + d) belong to donor B. Dots represent threonine-monophosphorylated ERK1/2, squares the tyrosine-monophosphorylated ERK1/2 and triangles diphosphorylated ERK1/2.

In the next step, we isolated peripheral blood mononuclear cells (PBMCs) from whole blood of two different healthy donors. The cells were stimulated with NMDA in the presence of glycine and analysed as described by the CIEF-immunoassay (Fig. 5). Maximum levels of approx. 2% of pY-ERK1 were observed 4 min after addition of NMDA (Fig. 5a,c). Maximum relative amounts of pY-ERK2 were even lower and were reached after 4 min NMDA treatment in donor A (Fig. 5b) and 2 min in donor B (Fig. 5d). The maximum signals of pp-ERK1 (5–7%) and pp-ERK2 (5–6%) were observed after 4 min treatment with NMDA and clearly exceeded those of pY-ERK1/2 and pT-ERK1/2 at this time point. Doubly phosphorylated pp-ERK1 decreased rapidly and was close to baseline levels 15 min after addition of NMDA (Fig. 5a,c). In contrast, for pp-ERK2 we observed a biphasic time course in both donors with a minimum after 8 min incubation followed by an increase. The pT-ERK1 and pT-ERK2-isoforms reached their maxima after 4–8 min followed by a comparatively slow decline. Monophosphorylated pT-ERK1 and pT-ERK2 were more abundant in stimulated cells than pY-ERK1 and pY-ERK2, respectively.

## Discussion

The detailed study of cellular activation and inactivation of ERK1/2 MAP kinases by dual phosphorylation and dephosphorylation at the conserved TEY activation motif is often achieved by Western blotting or ELISA-type assays involving the use of phosphorylation dependent antibodies. In a recent publication, Prabakaran and colleagues presented a sophisticated combined mass spectroscopy (MS) strategy for quantifying phospho-form distributions of multiply phosphorylated cellular proteins including ERK, without the need for specific antibodies^16^. Isoelectric focusing in microcapillaries followed by photochemical immobilisation and immunological detection with chemiluminescent readout represents a highly sensitive approach allowing for increased throughput. Additionally, the use of a single pan-specific and phosphorylation-insensitive anti ERK1/2 antibody enables the straightforward relative semi-quantification of diphosphorylated, monophosphorylated and unphosphorylated forms of ERK1/2 in minute samples^15^.

Prompted by the observation that in our hands monophosphorylated ERK1 and ERK2 regularly appeared as double peaks in the CIEF-electropherograms, we set out to further characterise the underlying charge isoforms of monophosphorylated ERK1 and ERK2. With the help of three different commercial antibodies against phosphorylated ERK1/2 and a series of synthetic phospho-peptides employed for competitive blocking experiments, we were able to unequivocally identify and differentiate Tyr- and Thr-monophosphorylated isoforms pT-ERK1, pY-ERK1, pT-ERK2 and pY-ERK2 in cell lysates from stimulated monocytic THP-1 and neuroblastoma SH-SY5Y cell lines and from human PBMCs prepared from whole blood. All three anti-phospho-ERK1/2 antibodies tested were competitively blocked by a synthetic peptide encompassing the TEY activation motif and being phosphorylated at both, threonine and tyrosine. The corresponding monophosphorylated pT-peptide erased the recognition of mono- and diphosphorylated ERK1/2 by antibody #4370, while the pY-peptide extinguished the detection of mono- and diphosphorylated ERK1/2 by antibodies #4377 and #040–477. Regarding antibodies #4370 and #4377 we have thus confirmed the information in terms of specificity provided in the accompanying data sheets of these commercial reagents. Taken together, the observations from the competitive blocking experiments suggest that all three tested anti-phospho ERK1/2 antibodies recognise ERK1/2 phosphorylated at either Thr (antibody #4370) or Tyr (antibodies #4377 and #040–477) irrespectively of the phosphorylation state of the remaining, second phosphorylation site within the TEY motif. Furthermore, our findings enabled the unequivocal assignment of the 8 different ERK-peaks detected by CIEF with a pan-specific antibody in activated cells to unphosphorylated, threonine-monophosphorylated, tyrosine-monophosphorylated and diphosphorylated forms of ERK1 and ERK2, respectively. The technology thus allows for an in-depth study of the ERK1/2 phospho-form distribution in cell lysates.

In SH-SY5Y cells stimulated with bradykinin, we observed a biphasic response of pp-ERK1, pp-ERK2, pT-ERK1 and pT-ERK2 with maxima after 6–8 and 12 min incubation time. Biphasic ERK1/2 activation with a similar, relatively quick time course was also reported by Asimaki and colleagues upon treatment of primary chicken embryo telencephalon neuronal cell cultures with the cannabinoid receptor 1 activator methanandamide^17^. However, the Western blot analysis applied in that study did not allow for a differentiation between monophosphorylated and diphosphorylated forms of ERK1/2.

The preliminary observations from our pilot experiments addressing ERK1/2 phosphorylation in response to NMDA stimulation in PBMC preparations from two different donors suggest that the relative distribution of pT-ERK1/2, pY-ERK1/2 and pp-ERK1/2 at different time points may differ substantially between different cell types and possibly also depending on the specific activator applied. Doubly phosphorylated pp-ERK1 and pp-ERK2 were maximal 4 min after addition of NMDA and then decreased relatively quickly, most likely due to the action of phosphatases. The monophosphorylated pT-ERK1 and pT-ERK2 reached higher levels in stimulated cells than pY-ERK1 and pY-ERK2, respectively. This was also the case in SH-SY5Y cells treated with bradykinin (Fig. 4).

The biological significance of monophosphorylated forms of ERK1/2 is currently not clear. Monophosphorylated ERK2 can result from phosphorylation by the upstream kinase MEK1 / MAPKK-1, which occurs by a non-processive, distributive mechanism^18^,^19^. Alternatively, partial dephosphorylation of fully activated pp-ERK2 by serine/threonine- or tyrosine specific phosphatases can generate the monophosphorylated pT-ERK2 and pY-ERK2, respectively^12^,^20^,^21^. The monophosphorylated forms of ERK2 were proposed to possibly represent intermediate activity states which might be linked to graded responses or might possess distinct biological functions^12^.

The ERK1/2 signalling cascade appears to be involved in diverse pathogenic processes, not only in oncogenesis but also in the context of Alzheimer’s disease, Parkinson’s disease and amyotrophic lateral sclerosis (ALS) (for reviews, see^9^,^10^). Furthermore, ERK1/2 has been proposed to represent a potential diagnostic biomarker in Alzheimer’s disease: Alterations in ERK1/2 phosphorylation in response to bradykinin have been observed in cultured skin fibroblasts from Alzheimer’s disease patients^22^,^23^,^24^, and elevated levels of total ERK1/2 protein in cerebrospinal fluid were reported^25^.

The data presented here may serve as a methodological basis for future in-depth studies addressing specific stimulus-dependent ERK1/2 phosphorylation / dephosphorylation kinetics in PBMCs, which have been proposed as a suitable material for dementia-related biomarker research (reviewed in^26^). These future experiments can be performed with a single pan-specific antibody and thus provide a direct semi-quantitative analysis of the relative abundances of all phospho ERK species in a single assay, including pT- and pY-ERK1/2. We speculate that these may turn out to be of relevance e.g. in the context of biomarker research in the fields of Alzheimer’s disease and cancer. Furthermore, the simultaneous semi-quantification of the different phosphorylated forms of ERK1/2 may facilitate studies aiming for an even better and more detailed understanding of the cellular regulation of ERK1/2 activity under physiological and pathophysiological conditions and may help to shed more light on the biological roles of the monophosphorylated forms of these kinases.

## Materials and Methods

### Isolation of peripheral blood mononuclear cells (PBMCs)

Signed informed consent was obtained from all volunteers, and the study procedures were approved by the ethics committee of the University of Duisburg-Essen. All methods were carried out in accordance with the approved guidelines. Whole blood was collected from healthy volunteers in 4 × 9 ml EDTA S-Monovettes (Sarstedt AG, Nümbrecht, Germany) and diluted 1:2 with washing buffer (PBS Dulbecco (Biochrom) containing 2 mM EDTA). Then, 12 ml Biocoll (density 1.077, Biochrom) were overlayed with 38 ml of the diluted blood in a 50 ml tube. After the gradient centrifugation step at 800 × g for 30 minutes at room temperature (RT) and with breaks turned off, the PBMCs were collected at the Biocoll/PBS-border, pooled and washed. The supernatant was discarded and the cell pellet resuspended in 10 ml of washing buffer. Following that, 10 ml FCS containing 5 mM EDTA were overlayed with the cell suspension and centrifuged at 80 × g for 15 minutes at RT without breaking. The cell pellet was washed and, finally, the PBMCs were resuspended in culture medium (RPMI 1640 media supplemented with 10% FCS, 10 mM HEPES (Life Technologies) and 100 U/ml penicillin/streptomycin (Life Technologies)) and counted on the Vi-Cell™ XR platform (Beckmann Coulter, Krefeld, Germany). Cells were adjusted to 5 × 10^6^ cells/ml, incubated for one night in a suspension culture flask (Greiner Bio One, Frickenhausen, Germany) in a humid environment at 37 °C and 5% CO_2_ and used for treatment experiments the following day.

### Cell culture

Human neuroblastoma cells (SH-SY5Y, ECACC/Sigma Aldrich, Taufkirchen, Germany) were cultured in DMEM + GlutaMAX™ (Life Technologies, Darmstadt, Germany) supplemented with 10% FCS (Biochrom, Berlin, Germany). Human monocytic cells (THP-1, ATCC/LGC Standards GmbH, Wesel, Germany) were cultured in RPMI 1640 media (Life Technologies) supplemented with 10% FCS and 0.05 mM beta-mercaptoethanol (Life Technologies). Both cell lines were kept at 37 °C in a humidified 5% CO_2_ atmosphere. SH-SY5Y cells were used for treatment experiments at 70% confluence and THP-1 cells at 0.8 × 10^6^ cells/ml.

### Cell stimulation

All three cell types were treated with 200 nM Phorbol 12-myristate 13-acetate (PMA, Sigma Aldrich). In addition, the SH-SY5Y cells were treated with 100 nM bradykinin (Calbiochem, Darmstadt, Germany). The PBMCs were treated with 50 μM N-Methyl-D-Aspartic Acid (NMDA, Sigma Aldrich) in combination with 10 μM glycine (Carl Roth, Karlsruhe, Germany). All substances were added to the cells in small volumes (≤1% of the final cell culture medium volume). To generate a time course of ERK 1/2 phosphorylation, stimulation was stopped at various time points by placing the cells on ice. According to the nature of the cells, the lysate preparation differed.

### SH-SY5Y lysate preparation

SH-SY5Y cells, were cultured in ∅6 cm dishes (TPP, Trasadingen, Switzerland). After the chosen stimulation time, the dish was placed on ice and the medium was discarded. Cells were washed once with washing buffer (PBS containing 2 mM EDTA), mechanically removed with a cell scraper (TPP) and transferred to a microreaction tube. The procedure was repeated once and the resulting cell suspension was then centrifuged at 500 × g for 5 minutes at 4 °C. The supernatant was discarded and 150 μl lysis buffer (M-PER reagent (Thermo Scientific/Pierce, Bonn, Germany) supplemented with 1x DMSO Inhibitor Mix (ProteinSimple, Santa Clara, CA, USA) and 1x Aqueous Inhibitor Mix (ProteinSimple)) were added. The cell pellet was resuspended by vortexing and incubated for 10 min at 4 °C on a shaker. The lysate was cleared by centrifugation at 16,000 × g for 15 min at 4 °C, aliquoted, quick-frozen in liquid nitrogen and stored at −80 °C until further applications. The protein concentrations were determined by bicinchoninic acid (BCA) assay (Thermo Scientific/Pierce).

### THP-1 and PBMC lysate preparation

1 ml aliquots of the suspension cells THP-1 and PBMCs were transferred to 15 ml tubes directly before the stimulation experiments. Following the chosen incubation times, the tubes were placed on ice and 10 ml ice cold washing buffer were added immediately. The cells were collected at 500 × g for 5 minutes at 4 °C. The cell pellet was then resuspended in 1 ml washing buffer, transferred to a microreaction tube and centrifuged again at 500 × g for 5 minutes at 4 °C. The supernatant was discarded and 50 μl lysis buffer were added to the cell pellets. They were dissolved by vortexing, shaken for 10 minutes at 4 °C and centrifuged at 16,000 × g for 15 minutes at 4 °C. The supernatants were then aliquoted, quick-frozen in liquid nitrogen and stored at −80 °C. The protein concentrations were determined by a bicinchoninic acid (BCA) assay (Thermo Scientific/Pierce).

### Capillary isoelectric focusing (CIEF) – immunoassay

The CIEF-immunoassay was performed on a NanoPro™ 1000 instrument (ProteinSimple) according to the manufacturer’s instructions. In brief, aliquots of the cell lysates were mixed with G2 separation premix (pH 5–8, nested), containing fluorescent pI standard ladder 3 (pI 4.9, 6.0, 6.4, 7.0, 7.3), an additional pI 5.5 standard and 1x DMSO–protease/phosphatase inhibitor mix. The final protein concentrations were 100 μg/ml and M-PER lysis buffer was used for volume adjustment. The sample mixtures were vortexed for 1 minute and centrifuged for 3 minutes at 16,000 × g at 4 °C. The supernatants, the primary and secondary antibodies and the luminol/peroxide XDR substrate mix were pipetted into a 384-well plate. All samples were analysed in technical duplicates. The phosphorylation-insensitive primary antibody PS#040-474 and the anti-phosphoERK1/2 antibody #040-477 (both obtained from ProteinSimple) were used without further dilution. The antibodies Phospho-p44/42 MAPK (Erk1/2) (Thr202/Tyr204) (D13.14.4E) XP® Rabbit mAb #4370 (Cell Signaling Technology/New England Biolabs GmbH, Frankfurt am Main, Germany) and Phospho-p44/42 MAPK (Erk1/2) (Thr202/Tyr204) (197G2) Rabbit mAb #4377 (Cell Signaling) were diluted 1:50 with antibody diluent (ProteinSimple) and the horseradish peroxidase (HRP)-conjugated secondary anti-rabbit IgG (Goat-Anti-Rabbit HUX Secondary Antibody, HRP-conjugate) was diluted 1:100 with antibody diluent (both provided by ProteinSimple). The plate was spun for 10 minutes at 1,500 × g at 4 °C and finally transferred to the NanoPro 1000 instrument.

The automated CIEF-immunoassay was programmed with the Compass software (ProteinSimple, version 1.3.7). Capillaries were filled with the sample mixture and isoelectric focusing electrophoresis was carried out at 21000 μW for 40 minutes. In the next step, the proteins and pI-standards were immobilised to the inner capillary wall by exposing to UV light for 100 seconds. Excess sample solution was washed away twice with wash buffer (load: 20 seconds, soak: 150 seconds) followed by incubation with the primary antibody for 120 minutes. After two further wash cycles the HRP-labeled anti-rabbit IgG secondary antibody was added for 60 minutes. Two more final wash steps were followed by the incubation with luminol/peroxide mix to generate chemiluminescent light which was recorded at different exposure times (30, 60, 120, 240, 480 and 960 seconds). Peak integration and pI marker calibration for peak alignment were performed with the Compass software. The percentage area of each phospho ERK1 and ERK2 isoform was calculated in relation to the added peak areas of all ERK1 and ERK2 peaks, respectively. Mean relative peak areas and standard deviations were calculated from technical duplicates.

### Competitive blocking with synthetic peptides for pT- and pY-ERK1/2 identification

For competitive blocking, we used lysates of PBMCs, which were treated with PMA to ensure a high phosphorylation level. The synthetic peptides KTGFL(pT)E(pY)VATR, KTGFL(pT)EYVATR (pT), KTGFL(pT)EYVATR (pY) and KTGFLTEYVATR (unphosphorylated) were kindly provided by Dr. Borek Vojtesek (Moravian Biotechnology Ltd (Brno, Czech Republic). The peptides correspond to the ERK1/2 amino acid sequence encompassing the conserved TEY activation motif at different phosphorylation states. An amino-terminal lysine residue (K) was added for coupling purposes in a different context. The four different peptides were mixed separately with each one of the three anti-phospho-ERK1/2 antibodies: #040–477 (ProteinSimple), #4370 (Cell Signaling) and #4377 (Cell Signaling) (see above). The phosphorylation-insensitive pan-ERK1/2 antibody (Rabbit; #040–474, ProteinSimple) served as a control. For the antibodies #4370 and #4377, 1 μl of the antibody stock solution was mixed with 44 μl antibody diluent and 5 μl of a peptide predilution, also prepared with antibody diluent. The final peptide concentrations in the assay were 10 μg/ml for the diphosphorylated-peptide, the pT-peptide and and the unphosphorylated peptide and 50 μg/ml for the pY-peptide. Regarding the phospho-ERK1/2 antibody #040–477 and the pan-ERK antibody (#040–474), 45 μl of the respective antibody solutions were directly mixed with 5 μl of the peptide predilution. The peptide-antibody-mixtures were applied to the CIEF-immunoassay as described above.

## Additional Information

**How to cite this article**: Kraus, I. *et al.* Detection and Differentiation of Threonine- and Tyrosine-Monophosphorylated Forms of ERK1/2 by Capillary Isoelectric Focusing-Immunoassay. *Sci. Rep.* **5**, 12767; doi: 10.1038/srep12767 (2015).

## Supplementary Material

Supplementary Information

## References

[b1] Cheung E. C. & Slack R. S. Emerging role for ERK as a key regulator of neuronal apoptosis. Science’s STKE : signal transduction knowledge environment 2004, PE45, 10.1126/stke.2512004pe45 (2004). 15383672

[b2] Cobb M. H. & Goldsmith E. J. How MAP kinases are regulated. The Journal of biological chemistry 270, 14843–14846 (1995). 7797459 10.1074/jbc.270.25.14843

[b3] Ramos J. W. The regulation of extracellular signal-regulated kinase (ERK) in mammalian cells. The international journal of biochemistry & cell biology 40, 2707–2719, 10.1016/j.biocel.2008.04.009 (2008). 18562239

[b4] Roskoski R. Jr ERK1/2 MAP kinases: structure, function, and regulation. Pharmacological research : the official journal of the Italian Pharmacological Society 66, 105–143, 10.1016/j.phrs.2012.04.005 (2012). 22569528

[b5] Wolf I. & Seger R. The mitogen-activated protein kinase signaling cascade: from bench to bedside. The Israel Medical Association journal : IMAJ 4, 641–647 (2002). 12183875

[b6] Adams J. P., Roberson E. D., English J. D., Selcher J. C. & Sweatt J. D. MAPK regulation of gene expression in the central nervous system. Acta neurobiologiae experimentalis 60, 377–394 (2000). 11016081 10.55782/ane-2000-1357

[b7] Grewal S. S., York R. D. & Stork P. J. Extracellular-signal-regulated kinase signalling in neurons. Current opinion in neurobiology 9, 544–553, 10.1016/S0959-4388(99)00010-0 (1999). 10508738

[b8] Sweatt J. D. The neuronal MAP kinase cascade: a biochemical signal integration system subserving synaptic plasticity and memory. Journal of neurochemistry 76, 1–10 (2001). 11145972 10.1046/j.1471-4159.2001.00054.x

[b9] Kim E. K. & Choi E. J. Pathological roles of MAPK signaling pathways in human diseases. Biochimica et biophysica acta 1802, 396–405, 10.1016/j.bbadis.2009.12.009 (2010). 20079433

[b10] Deschenes-Simard X., Kottakis F., Meloche S. & Ferbeyre G. ERKs in cancer: friends or foes? Cancer research 74, 412–419, 10.1158/0008-5472.CAN-13-2381 (2014). 24408923

[b11] Payne D. M. *et al.* Identification of the regulatory phosphorylation sites in pp42/mitogen-activated protein kinase (MAP kinase). The EMBO journal 10, 885–892 (1991). 1849075 10.1002/j.1460-2075.1991.tb08021.x PMC452730

[b12] Zhou B. & Zhang Z. Y. The activity of the extracellular signal-regulated kinase 2 is regulated by differential phosphorylation in the activation loop. The Journal of biological chemistry 277, 13889–13899, 10.1074/jbc.M200377200 (2002). 11839761

[b13] Gribble F. M. *et al.* A novel method for measurement of submembrane ATP concentration. The Journal of biological chemistry 275, 30046–30049, 10.1074/jbc.M001010200 (2000). 10866996

[b14] Cha H. & Shapiro P. Tyrosine-phosphorylated extracellular signal--regulated kinase associates with the Golgi complex during G2/M phase of the cell cycle: evidence for regulation of Golgi structure. The Journal of cell biology 153, 1355–1367 (2001). 11425867 10.1083/jcb.153.7.1355 PMC2150730

[b15] O’Neill R. A. *et al.* Isoelectric focusing technology quantifies protein signaling in 25 cells. Proceedings of the National Academy of Sciences of the United States of America 103, 16153–16158, 10.1073/pnas.0607973103 (2006). 17053065 PMC1618307

[b16] Prabakaran S. *et al.* Comparative analysis of Erk phosphorylation suggests a mixed strategy for measuring phospho-form distributions. Molecular systems biology 7, 482, 10.1038/msb.2011.15 (2011). 21487401 PMC3097084

[b17] Asimaki O. & Mangoura D. Cannabinoid receptor 1 induces a biphasic ERK activation via multiprotein signaling complex formation of proximal kinases PKCepsilon, Src, and Fyn in primary neurons. Neurochemistry international 58, 135–144, 10.1016/j.neuint.2010.11.002 (2011). 21074588

[b18] Ferrell J. E. Jr & Bhatt R. R. Mechanistic studies of the dual phosphorylation of mitogen-activated protein kinase. The Journal of biological chemistry 272, 19008–19016 (1997). 9228083 10.1074/jbc.272.30.19008

[b19] Burack W. R. & Sturgill T. W. The activating dual phosphorylation of MAPK by MEK is nonprocessive. Biochemistry 36, 5929–5933, 10.1021/bi970535d (1997). 9166761

[b20] Alessi D. R. *et al.* Inactivation of p42 MAP kinase by protein phosphatase 2A and a protein tyrosine phosphatase, but not CL100, in various cell lines. Current biology : CB 5, 283–295 (1995). 7780739 10.1016/s0960-9822(95)00059-5

[b21] Saxena M., Williams S., Brockdorff J., Gilman J. & Mustelin T. Inhibition of T cell signaling by mitogen-activated protein kinase-targeted hematopoietic tyrosine phosphatase (HePTP). The Journal of biological chemistry 274, 11693–11700 (1999). 10206983 10.1074/jbc.274.17.11693

[b22] Khan T. K. & Alkon D. L. An internally controlled peripheral biomarker for Alzheimer’s disease: Erk1 and Erk2 responses to the inflammatory signal bradykinin. Proceedings of the National Academy of Sciences of the United States of America 103, 13203–13207, 10.1073/pnas.0605411103 (2006). 16920798 PMC1559777

[b23] Khan T. K. & Alkon D. L. Early diagnostic accuracy and pathophysiologic relevance of an autopsy-confirmed Alzheimer’s disease peripheral biomarker. Neurobiology of aging 31, 889–900, 10.1016/j.neurobiolaging.2008.07.010 (2010). 18760507

[b24] Zhao W. Q. *et al.* MAP kinase signaling cascade dysfunction specific to Alzheimer’s disease in fibroblasts. Neurobiology of disease 11, 166–183 (2002). 12460556 10.1006/nbdi.2002.0520

[b25] Spitzer P. *et al.* Evidence for Elevated Cerebrospinal Fluid ERK1/2 Levels in Alzheimer Dementia. International journal of Alzheimer’s disease 2011, 739847, 10.4061/2011/739847 (2011). PMC3227514 22145083

[b26] Mandas A. & Dessi S. Mononuclear cells in dementia. Clinica chimica acta; international journal of clinical chemistry 431, 278–287, 10.1016/j.cca.2014.02.016 (2014). 24582859

