# Supplementary material for: Detection and Differentiation of Threonine- and Tyrosine-Monophosphorylated Forms of ERK1/2 by Capillary Isoelectric Focusing-Immunoassay
Source: Sci Rep. 2015 Aug 3;5:12767. doi: 10.1038/srep12767 (PMC4522687; doi:10.1038/srep12767)
Supplement: Supplementary Information [file srep12767-s1.pdf]

Supplementary Information to:

Detection and Differentiation of Threonine- and Tyrosine-Monophosphorylated Forms of ERK1/2 by Capillary Isoelectric Focusing-Immunoassay

Inga Kraus[1,2,5], Daniela Besong Agbo[1], Markus Otto[3], Jens Wiltfang[4,2], Hans Klafki[1,5*]

[1] LVR-Hospital Essen, Department of Psychiatry and Psychotherapy, Faculty of Medicine, University of Duisburg-Essen, Essen, Germany

[2] German Center for Neurodegenerative Diseases (DZNE), Research Site Goettingen, Germany

[3] Department of Neurology, University of Ulm, Germany

[4] Dept. of Psychiatry and Psychotherapy, University Medical Center Goettingen (UMG), Georg-August-University Goettingen, Germany

[5] Current address: Dept. of Psychiatry and Psychotherapy, University Medical Center Goettingen (UMG), Georg-August-University Goettingen, Germany

*Corresponding author

Hans.Klafki@med.uni-goettingen.de

Peak Identification by Different Commercial ERK Specific Antibodies for CIEF-immunoassay

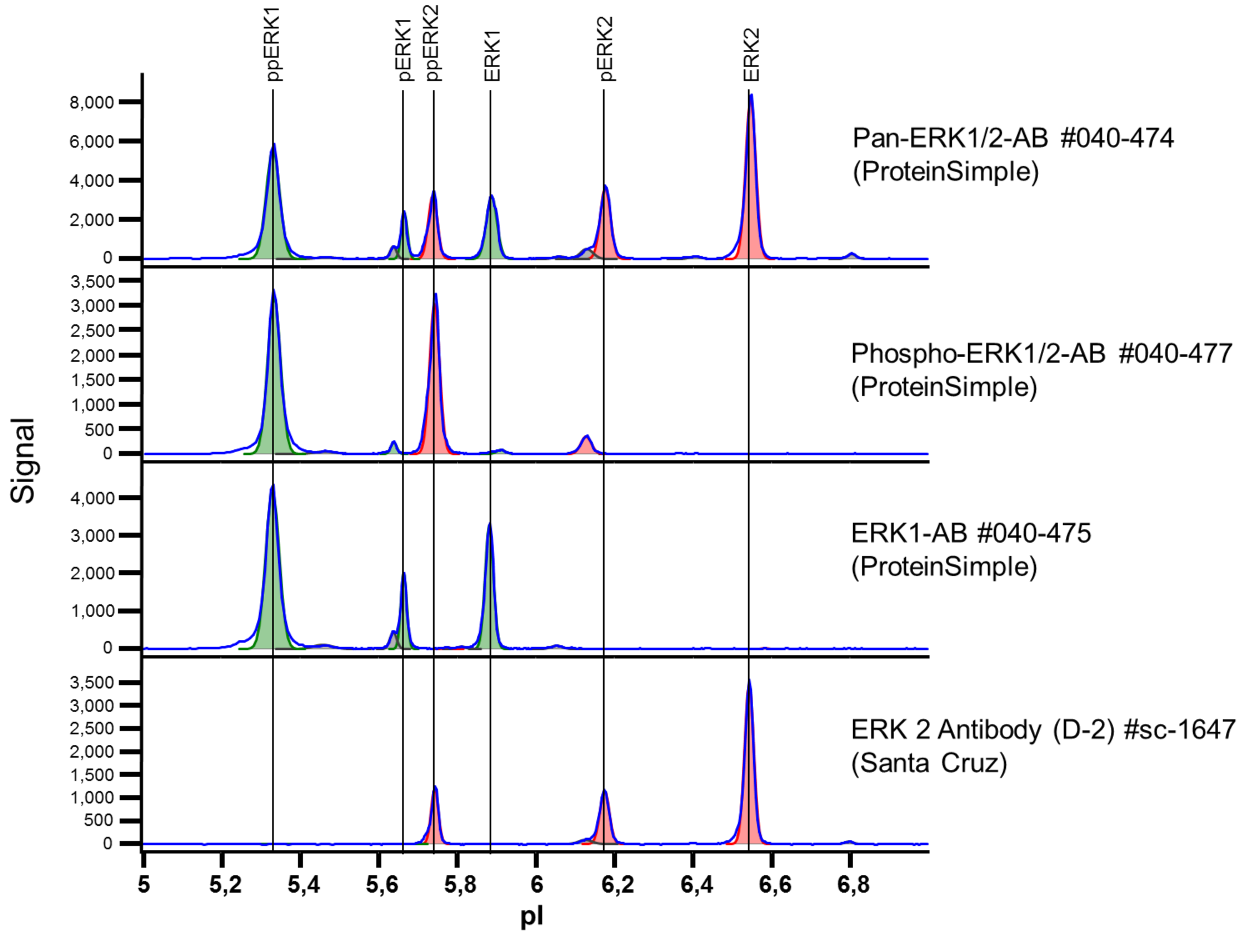

Figure S1: Identification of unphosphorylated-, mono- and diphosphorylated ERK1 and ERK2 isoforms. Human PBMCs derived from whole blood were treated with 200 nM PMA and lysed after 10 min. Aliquots of the lysates were subjected to CIEF-immunoassay. The pan-ERK1/2-antibody (#040-474, ProteinSimple, Santa Clara , California, USA), the phospho-ERK1/2-antibody (#040-477, ProteinSimple) and the ERK1-antibody (#040-475, ProteinSimple) were applied as described under materials and methods. The monoclonal mouse ERK2 antibody (D-2) #sc-1647 (Santa Cruz Biotechnology, Dallas, Texas, USA) was diluted 1:50 with antibody diluent and used in combination with a HRP-conjugated Goat-Anti-Mouse Secondary antibody (#040-655, ProteinSimple), which was diluted 1:100 with antibody diluent. The phosphorylation-insensitive pan-ERK1/2 antibody detects all ERK1/2 isoforms within this sample. The selective anti-phoshoERK1/2, anti-ERK1 and anti-ERK2 antibodies allow for unequivocal assignment of the different peaks. ERK1 isoforms are displayed in green, ERK2 isoforms in red.
